# Knowledge of Medical Students Towards the Re-emergence of Human Monkeypox Virus

**DOI:** 10.7759/cureus.46761

**Published:** 2023-10-09

**Authors:** Hemalatha Raman, Aslinda Jamil, Abdur Rasheed, Ammar Abdulrahman Jairoun, Pei Lin Lua, Umar Idris Ibrahim, Shazia Jamshed

**Affiliations:** 1 Faculty of Pharmacy, Universiti Sultan Zainal Abidin, Terengganu, MYS; 2 School of Public Health, Dow University of Health Sciences, Karachi, PAK; 3 Health and Safety, Centre of Medical and Bio-allied Health Sciences Research, Ajman University, Dubai, ARE

**Keywords:** re-emergence, outbreak, medical students, knowledge level, hmpx

## Abstract

Introduction: Declaration of human monkeypox(HMPX) virus as Public Health Emergency of International Concern (PHEIC) by World Health Organisation (WHO) has raised concerns among the public andlack of knowledge is a prominent challenge in curbing this outbreak. Therefore, assessment ofknowledge level on this outbreak among the medical students is also necessary due to the fact that they are the future healthcare practitioners who will be directly involved in the disease management as well as a major source of knowledge dissemination to the public.

Aim: The main objective of this study is to assess the knowledge level of medical students at Universiti Sultan Zainal Abidin (UniSZA) regarding the emergence of HMPX. Additionally, the study aims to investigate potential associations between socio-demographic characteristics and knowledge levels, while also identifying factors that predict a high level of knowledge in this context..

Methods: A descriptive cross-sectional study was conducted among UniSZA undergraduatemedical students from Year 1 to Year 5. A validated questionnaire comprising six socio-demographic variables and 27 knowledge items was shared online. Descriptive statistics, non-parametric tests and multivariate logistic regression were performed using SPSS software.

Results: A total of 138 medical students out of 300 participated in this study. Overall, the average knowledge score was 73.95% ±4.43, which indicates that the medical students have moderate knowledge level. Nearly half of them had good knowledge level (n= 68; 49.3%), 43 of them had moderate knowledge level (31.2%), and 27 of them had poor knowledge level (19.6%). There was a significant association between knowledge level and two factors: receiving information on HMPX during their education and seniority (P-value < 0.01 and P-value < 0.05, respectively). Besides, received information on HMPX during their education was a significant predicting factor of good knowledge level (P-value = 0.002).

Conclusion: The knowledge level among the medical students was relatively inadequate.

## Introduction

Human monkeypox (HMPX) virus is a viral zoonotic virus caused by a monkeypox virus (MPXV), which is a genus species of Orthopoxvirus of the family Poxviridae [1]. Upon the eradication of chickenpox virus infection, HMPX has steadily emerged with constantly increasing number of cases across Africa and other countries such as the UK, the US, and Singapore [2]. It is reported that it possibly spread through aerosol transmission upon nosocomial outbreak in the Central African Republic [3]. With regard to the transmission route, this virus can transmit mainly from animals to humans or from humans to humans. In addition, it also may spread from mother to foetus, through usage of contaminated items of an infected individual, and possibly through sexual transmission [4].

As for the clinical presentation, the most signifying symptom is the swollen lymph nodes or also known as lymphadenopathy. In addition, the occurrence of fever, headache, blister-like rash, and fatigue is often misdiagnosed with chickenpox. However, it should be also noted that HMPX is less severe and less fatal compared to chickenpox [5]. Besides lymphadenopathy, one of the common differences between these two viruses is their features of sores. HMPX’s sores are usually deeper and more painful compared to chickenpox’s sores that are small in size and itchy. The lasting effect of those who survive HMPX is pitted scarring [6].

In addition, the treatment regimen is still not developed specifically for HMPX. However, anti-viral medications used to treat chickenpox is also used for HMPX as well. Currently, the antiviral drug tecovirimat (TPOXX) has been approved by the US Food and Drug Administration (FDA) to treat severe HMPX cases in adults and children. However, this drug is still an investigational drug. Research is currently being conducted to test the safety and effectiveness for all people with HMPX [7].

The growing number of HMPX cases illustrates the importance of early detection, management as well as prevention of HMPX majorly from the healthcare authorities [8]. In addition, World Health Organization (WHO) added that lack of knowledge is the major challenge in tackling this outbreak. In accordance with that, assessment of knowledge level among the medical students is necessary since they are the future healthcare professionals who are widely responsible for disease management and treatment recommendation. Lack of knowledge can be a drawback for them since there is a high possibility for misdiagnosis with chickenpox, inappropriate treatment recommendation and preventive measures.

Thus, the aim of this study is to evaluate the knowledge level of medical students on the re-emergence of HMPX. Furthermore, this study is intended to determine the association between the socio-demographic characteristics and knowledge level and evaluate the predicting factors that contribute to good knowledge level.

## Materials and methods

Study design

This study was a descriptive cross- sectional study of medical students’ knowledge level towards the HMPX outbreak. This study was conducted among University Sultan Zainal Abidin (UniSZA) undergraduate medical students from year 1 until year 5. The data was collected from 24 March until 1 June 2023. Data were collected via an anonymous online questionnaire. Approval of the study was obtained from Human Research Ethics Committee of University Sultan Zainal Abidin (study protocol code: UniSZA/UHREC/2023/481).

Participants

All UniSZA undergraduate medical students who have enrolled in Bachelor of Medicine& Surgery (MBBS) program are eligible to participate in this study. Students enrolled in any different program other than MBBS or/and studying in universities besides UniSZA as well as those medical students who are unwilling to participate are excluded from this study. The total number of medical students was 300, and they were further classified into seniors and juniors. The term 'juniors' is used to represent preclinical students who are 1st, 2nd and 3rd year whereas the term 'seniors' is used to represent 4th and 5th year clinical students.

Study instrument

The study questionnaire was adapted from a validated study that similarly investigated knowledge level of HMPX re-emergence among the Al Ain University students in Dubai [8]. The questionnaire was then created on Google Form. It comprised of two sections, which had six items on socio-demographic characteristics and 27 knowledge items testing their knowledge level on HMPX's source, signs and symptoms, treatment, preventive measures and mode of transmission. Respondents were given three options to choose from which are “Yes”, “No” or “I don’t know”. It was mandatory for the respondents to answer all the items to submit a valid response.

Study procedure

Links of the developed questionnaire were sent simultaneously via various social media platforms such as Telegram, WhatsApp, and Instagram. Each medical student was eligible to respond to the online questionnaire only once.

Data analysis

Data were analysed on Statistical Package for Social Sciences (SPSS) version 25 to evaluate the knowledge level of medical students. Prior to analysis, the knowledge scoring was done. Respondents were given a score of '1' for correct response and '0' for incorrect or 'I don't know' response. Knowledge domain comprising of 21 items were assessed to determine the knowledge level of medical students. The score was totaled up to produce a total score ranging from 0 to 21. The obtained total score was converted into percentage to determine the participants’ knowledge level. Bloom’s cutoff criteria was used to classify the score into good, moderate, and poor knowledge level [9]. Accordingly, participants' overall knowledge of HMPX was classified as good if the score is between 80% and 100% (17-21 points), moderate for a score between 60% and 79% (13-16.5 points), and poor for a score below 60% (< 13 points). Using SPSS software, descriptive statistics were summarized as frequency percentages and continuous variables were summarized using mean SD. Univariate and multivariate analysis of factors associated with the good knowledge about HMPX were implied to determine the influencing factors of good knowledge level.All variables that have p-value < 0.25 in the univariate analysis were included in the regression multivariate analysis. The regression model reasonable fitted well and all assumptions were met. No interactions and multicollinearity problems were identified. The statistical significance level was set at p < 0.05.

## Results

Table 1 shows the responses for socio-demographic characteristics of the participants. Out of 300 medical students, 138 of them participated in this study thus yielding a response rate of 46%. The mean age of the participants was 22.27 SD of 1.478 years. Majority of the participants were female, 82 (59.4%), and more than half were juniors 87 (63%). Furthermore, most prevalent source of information was social media 96 (69.6%).

**Table 1:** Socio-demographic data presented as frequencies and percentages

Demographics		Frequency (n)	Percentage (%)
Age	22.27 ± 1.478		
Gender	Male	56	40.6
	Female	82	59.4
Have you ever get infected by human chickenpox?	No	67	48.6
	Yes	71	51.4
Had you ever received information of Human Monkeypox virus during education?	No	52	37.7
	Yes	86	62.3
Source of information about Human Monkeypox virus:	Social media	96	69.6
	Television	13	9.4
	Awareness campaign	11	8
	Friends & family	18	13
Seniority	Junior	87	63
	Senior	51	37

**Figure 1:**
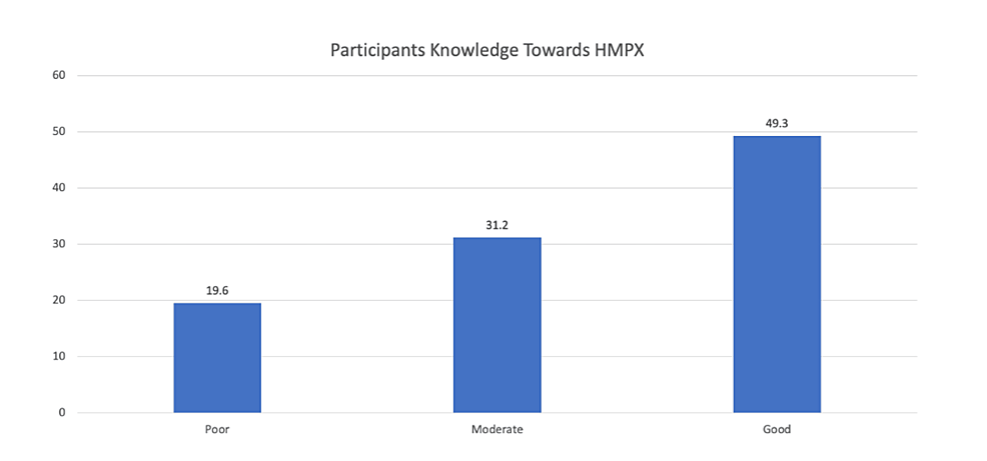
Classification of participants' knowledge level

The average knowledge score was 15.5 with a 95% CI of 14.78 to 16.27. Based on Figure 1, it is evident that nearly 50% of the medical students have a good knowledge level (49.3%), followed by moderate (31.2%) and poor (19.6%) knowledge levels .

Table 2 shows the responses for each question, which are classified into correct and incorrect. Nearly all the participants were aware that HMPX is a viral disease not bacterial disease (129, 93.5%). In addition, more than half were aware that HMPX and chickenpox share similar symptoms (95, 68.8%). In addition, three-quarter of the students correctly answered question on the signs and symptoms of the HMPX. Furthermore, most differentiating symptom of HMPX from chickenpox which is lymphadenopathy was correctly answered by more than half of the students (85, 61.6%). As for the preventive measures, an overall satisfactory level was observed in which more than half of the students agreed that symptomatic supportive care is one of the management options for HMPX (103, 74.6%). However, most of the treatment items were incorrectly answered by the students. For example, almost all the students incorrectly understood that the HMPX can be treated with the anti-viral medication (117, 84.8%). They were also unaware of the unavailability of treatment for HMPX until now (42, 58%).

**Table 2:** Knowledge items about HMPX source, signs/symptoms, transmission, prevention and treatment HMPX: Human Monkeypox

		Correct response	Correct answer		Incorrect answer	
			n	%	n	%
1	Monkeypox is a viral disease infection	Yes	129	93.5	9	6.5
2	Monkeypox is a bacterial disease infection	No	111	80.4	27	19.6
3	Monkeypox occurs in primarily in tropical rainforest areas of Africa and is occasionally exported to other regions	Yes	94	68.1	44	31.9
4	Monkeypox and chickenpox have similar signs and symptoms	Yes	95	68.8	43	31.2
5	The interval from infection to onset of symptoms is usually from 6 to 13 days but can range from 5 to 21 days	Yes	68	49.3	70	50.7
6	Monkeypox is easily transmitted	Yes	106	76.8	32	23.2
7	Monkeypox is transmitted from animal-to-human, through direct contact with the blood, bodily fluid, cutaneous or mucosal lesions of infected animal or eating insufficiently cooked meat from an infected animal	Yes	111	80.4	27	19.6
8	Flu-like syndrome is one of the early signs or symptoms of Human Monkeypox	Yes	97	70.3	41	29.7
9	Skin rashes usually begin within 1-3 days of fever and are one of the signs or symptoms of Human Monkeypox	Yes	105	76.1	33	23.9
10	Papules on the skin are one of the signs or symptoms of Human Monkeypox	Yes	100	72.5	38	27.5
11	Vesicles on the skin are one of the signs or symptoms of Human Monkeypox	Yes	86	62.3	52	37.7
12	Pustules on the skin are one of the signs or symptoms of Human Monkeypox	Yes	83	60.1	55	39.9
13	Lymphadenopathy (swollen lymph nodes) is one clinical sign or symptom that could be used to differentiate Monkeypox and chickenpox case	Yes	85	61.6	53	38.4
14	Fever, exhaustion, back and muscle ache, and intense headaches are the signs or symptoms of Human Monkeypox	Yes	112	81.2	26	18.8
15	Frequent hands washing for at least 20 seconds with soap and water- or alcohol-based hand sanitizers is essential to prevent further transmission	Yes	113	81.9	25	18.1
16	Avoiding contact with wild animals (alive or dead) is essential to prevent further transmission	Yes	112	81.2	26	18.8
17	Monkeypox could be prevented by cooking meat properly	Yes	80	58	58	42
18	Avoiding contact with any objectives that have been in contact with sick animal can prevent spread of disease	Yes	114	82.6	24	17.4
19	Avoiding contact with any person that has a rash can prevent spread of disease	Yes	110	79.7	28	20.3
20	Avoiding contact with any objective that has been in contact with sick person can prevent spread of disease	Yes	106	76.8	32	23.2
21	Reporting symptoms of Monkeypox to local health authorities is important to prevent further disease transmission	Yes	126	91.3	12	8.7
22	Monkeypox usually a self-limited disease with the symptoms lasting from 2 to 4 weeks	Yes	73	52.9	65	47.1
23	Symptomatic supportive care is to be considered in the management of Monkeypox disease	Yes	103	74.6	35	25.4
24	One management option for Monkeypox patients who are symptomatic is to use paracetamol	Yes	67	48.6	71	51.4
25	Antibiotics are effective in HMPX treatment	No	70	50.7	68	49.3
26	Monkeypox can be treated with available antiviral medications	No	21	15.2	117	84.8
27	There is no treatment until now	Yes	58	42	80	58

Table 3 depicts the association between socio-demographic variables and the knowledge level. As per the findings, there was a significant association between knowledge level and two factors: receiving information on HMPX during their education and seniority (P-value < 0.01 and P-value < 0.05, respectively). Thus, students who have received information had a better knowledge level (16.53) compared to those who did not (13.86), whereas the seniors had a significantly better knowledge level (15.94) compared to juniors (15.28).

**Table 3:** Participants' knowledge towards HMPX virus according to demographics HMPX: Human Monkeypox Chi-square test for relatedness was performed. Statistical significance was set at p <0.05.

Demographic		Mean	±SD	P-value
Gender	Male	15.62	4.68	4.98
	Female	15.46	4.27	
Have you ever get infected by human chickenpox?	No	14.94	4.81	0.168
	Yes	16.08	3.98	
Had you ever received information of Human Monkeypox virus during education?	No	13.86	4.44	<0.0001*
	Yes	16.53	4.12	
Source of information about Human Monkeypox virus:	Social media	15.25	4.63	0.445
	Television	16	4.2	
	Awareness campaign	17.36	2.83	
	Friends & family	15.55	4.21	
Seniority	Junior	15.28	3.63	0.014*
	Senior	15.94	5.54	

Furthermore, Table 4 shows the predicting factors among the socio- demographic characteristics that influence the good knowledge level. Based on multivariate analysis, it was found that receiving information on HMPX virus during education (OR 3.221; 95% CI 1.527-6.797) was the only significant predicting factor for a good knowledge level. Therefore, students who have received information are 3.22 times more likely to have significantly better knowledge level than those who did not receive information.

**Table 4:** Univariate and multivariate analysis of factors associated with the good knowledge about HMPX α < 0.05

Univariate						Multivariate		
	OR	95% CI Lowerbound	Upper bound	P- value	OR	95%CI Lower bound	Upper bound	P-value
Age	1.276	1.008	1.614	0.042	0.984	0.672	1.44	0.933
Gender (Ref: Male)								
Female	0.748	0.379	1.479	0.405	-	-	-	-
Seniority (Ref: Junior)								
Senior	2.724	1.33	5.577	0.006	2.605	0.814	8.342	0.107
Have you ever get infected by human chickenpox? (Ref:No)								
Yes	1.264	0.647	2.467	0.493	-	-	-	-
Had you ever received information of Human Monkeypox virus during education? (Ref: No)								
Yes	3.441	1.657	7.144	0.001	3.221	1.527	6.797	0.002
Source of information about Human Monkeypox virus (Ref:social media)								
Television	1.438	0.45	4.597	0.54	-	-	-	-
Awareness campaign	12.326	1.517	100.112	0.019	-	-	-	-
Friends & family	0.986	0.358	2.715	0.978	-	-	-	-

## Discussion

Due to the major concern regarding this outbreak among the public, effective and proactivemeasures need to be taken. The management of this outbreak requires adequate knowledgeespecially among the medical students due to their prominent role as future healthcarepractitioners who will be directly involved in the clinical care of the public. Therefore, this study hasevaluated the knowledge level among the UniSZA medical students on the re-emergence of HMPX.

Overall, the knowledge score among medical students was found to be at a moderate level, indicating that these students possess an insufficient level of knowledge regarding HMPX. This moderate knowledge level can be attributed to the absence of actual HMPX cases reported in Malaysia. Despite the recent global concerns surrounding HMPX outbreaks, Malaysia has not recorded any cases, in contrast to the outbreaks of other infectious diseases like the Covid-19 pandemic. A similar observation was made in a study by Gonzales-Zamora et al., where physicians in Peru exhibited a remarkably high median knowledge score of 14 (with an interquartile range of 13 to 15) [10]. This result was attributed to Peru being among the countries with a relatively high number of reported HMPX cases, ranking 7th globally. Hence, exposure to actual cases of an infectious disease appears to significantly influence participants' knowledge levels.

However, these findings contrast with those reported by Gonzales-Zamora et al. and Ugwu et al., where participants demonstrated a good knowledge level [10,11]. This difference was attributed to the use of informative infographics during their educational periods."

An analysis of the data revealed that higher knowledge levels were observed among male students, seniors, individuals with a history of chickenpox, and those who received information about HMPX during their educational courses. Several factors may account for these differences. Firstly, increased media coverage of the re-emergence of this disease, especially through social media channels, may have contributed to the better awareness among medical students.

Additionally, seniors displayed a superior knowledge level, possibly owing to their exposure to clinical settings with frequent patient interactions compared to juniors, who tend to have a more theory-oriented focus in their education. Furthermore, individuals who received information about HMPX exhibited a more robust knowledge base, as they would have been exposed to epidemiological and pathophysiological aspects of HMPX more extensively compared to their counterparts who did not receive such information. 

These findings are consistent with a study by Alshahrani et al., which emphasized that providing students with information about HMPX can enhance their confidence in dealing with the disease [12]. Furthermore, a significant percentage of students identified social media as their primary source of information. However, it is noteworthy that no significant association was established between the source of information and the level of knowledge. The mean age of the students in this study was 22 years, which justifies their preference for social media as a primary information source, given its widespread use among younger populations.

Despite the extensive media coverage of HMPX and the awareness it has generated among medical students, it is crucial to emphasize that the choice of information source did not correspond proportionally with their knowledge level. This discrepancy may be attributed to the potential lack of reliable and accurate information on social media platforms. In many cases, social media can be a source of misinformation and the propagation of myths, which might have influenced the students' knowledge levels in an adverse manner.

When considering the knowledge assessment items, it is noteworthy that medical students performed well across various domains, including questions related to sources/definitions, mode of transmission, preventive measures, and signs and symptoms of HMPX. The higher percentage of correct responses observed in this current study, in comparison to previous studies by Alshahrani et al. and Harapan et al., can be attributed to several factors [12,13]. These factors include the information imparted to students during their formal education and the use of social media as a supplementary source of information.

Significantly, students demonstrated a commendable ability to differentiate between chickenpox and HMPX, with a substantial majority correctly identifying lymphadenopathy as the primary distinguishing symptom from chickenpox. This distinction is of paramount importance given the frequent misdiagnosis of HMPX and chickenpox due to the similarity of other symptoms, such as fever, fluid-filled sores, cropping rash, myalgia, and arthralgia [14,15]. Consequently, accurate diagnosis based on a sound understanding of the distinctive signs and symptoms can potentially enhance the quality of life for patients by facilitating precise diagnosis and the selection of appropriate treatment options [16].

However, it is important to note that students exhibited a notable deficiency in their knowledge related to treatment aspects. While treatment items were not factored into the assessment of the overall knowledge score, evaluating their understanding of treatment could provide valuable insights into knowledge gaps. Over half of the participants mistakenly believed that antibiotics are effective in the treatment of HMPX. This misconception about treatment can pose a significant risk to patients, as it may lead to inappropriate treatment regimens being chosen.

Therefore, it is recommended that the incorporation of teaching related to basic tropical medicine and the treatment of infectious diseases be considered to enhance students' comprehension of disease identification and treatment, ensuring appropriate therapeutic choices [17]. Additionally, it is noteworthy that a majority of students disagreed with the statement that "there is no available treatment for HMPX" and believed it could be treated with existing antiviral medications. However, in reality, there is no specific antiviral medication available for HMPX, and the treatment used for chickenpox is employed in HMPX cases due to their similarity in terms of nucleoprotein (as recommended by WHO and CDC). This misconception regarding available antiviral medication may be attributed to confusion between the treatment of chickenpox and HMPX.

To address this, it is advisable to implement more vaccination campaigns and enhance the focus on the treatment of emerging diseases within the curriculum. This would help ensure that students have a thorough understanding of disease management, particularly in cases where specific antiviral treatments may not be available.

Furthermore, this study conducted an assessment of predictive factors for achieving a high level of knowledge. Notably, the item 'have received information on HMPX during the period of education' emerged as the sole significant determinant for attaining a high level of knowledge. This finding underscores the potential for students to augment their theoretical understanding of this disease through education, even in the absence of direct exposure to real cases.

There are some limitations in this study. First of all, this study is prone to selection bias due to the convenience sampling and data collection procedure which was conducted online. Additionally, academic dishonesty is a possibility since the students would have surf the internet to obtain the accurate information for the knowledge items. 

## Conclusions

As a conclusion, medical students had an average knowledge level on the re-emergence of HMPX. A significant higher knowledge level was found among seniors and students who had received information during education. Most of the students responded well on the mode of transmission, prevention, signs, and symptoms, but a knowledge gap was identified in the treatment items that could be due to the confusion between the treatment of HMPX and chickenpox. Therefore, it is suggested that holding webinars, conferences, awareness campaigns and further emphasize on infectious disease in their curriculum could fill the knowledge gap and potentially help in improving their knowledge level and understanding.
